# Niclosamide induced cell apoptosis via upregulation of ATF3 and activation of PERK in Hepatocellular carcinoma cells

**DOI:** 10.1186/s12876-016-0442-3

**Published:** 2016-02-25

**Authors:** Shunyan Weng, Liang Zhou, Qing Deng, Jiaxian Wang, Yan Yu, Jianwei Zhu, Yunsheng Yuan

**Affiliations:** Engineering Research Center of Cell and Therapeutic Antibody, Ministry of Education, School of Pharmacy, Shanghai Jiao Tong University, 800 Dongchuan Rd, Biology and Pharmacy Building, suite 6-208, Shanghai, 200240 People’s Republic of China; The Shanghai Municipality Key Laboratory of Veterinary Biotechnology, School of Agriculture and Biology, Shanghai Jiao Tong University, 800 Dongchuan Rd, Shanghai, 200240 People’s Republic of China; School of Life Sciences and Biotechnology, Shanghai Jiao Tong University, 800 Dongchuan Rd, Shanghai, 200240 People’s Republic of China

**Keywords:** Activating transcription factor 3, Endoplasmic reticulum stress, Reactive oxygen species, Liver cancer

## Abstract

**Background:**

Hepatocellular carcinoma (HCC) is one of most common and aggressive human malignancies in the world, especially, in eastern Asia, and its mortality is very high at any phase. We want to investigate mechanism of niclosamide inducing cell apoptosis in HCC.

**Methods:**

Two hepatoma cell lines were used to evaluate activity of niclosamide inducing cell apoptosis and study its mechanism. Quantitative real-time PCR and western blotting were used in analysis of genes expression or protein active regulated by niclosamide.

**Results:**

Niclosamide remarkably induced cell apoptosis in hepatoma cells. Furthermore, our study revealed that RNA-dependent protein kinase-like kinase (PERK) is activated and its expression is up-regulated in HCC cells which are exposed to niclosamide. niclosamide also significantly increase activating transcription factor 3 (ATF3), activating transcription factor 4 (ATF4) and CCAAT/enhancer-binding protein-homologous protein (CHOP) expression in HCC cells. It’s suggested that the function of niclosamide was abrogated by PERK inhibitor or absent ATF3. Expression of PERK and CHOP is correlated with ATF3 level in the cells.

**Conclusion:**

Taken together, our results indicate that ATF3 plays an integral role in ER stress activated and cell apoptosis induced by niclosamide in HCC cells. In this study, the new mechanism of niclosamide as anti-cancer we investigated, too.

## Background

Hepatocellular carcinoma (HCC) is one of the most common and aggressive human malignancies in the world [1]. It is a major public health issue worldwide according to epidemiological data, and the incidence is high in East Asia.

HCC often develops caused by chronic tissue damage due to liver cirrhosis which could be induced by HBV, HCV, alcohol intake, hemochromatosis, nonalcoholic steatohepatits and so on [2].

Surgical resection is the primary treatment option for patients with early stage of HCC [2]. After surgical treatment, patients should accept chemotherapy. However the rate of total 5-years survival is very low due to side effects of chemotherapeutic drugs and the chemo-resistance of tumor cells. Therefore it is very important to develop new drugs for HCC treatment [3]. It is well known that Endoplasmic Reticulum (ER) stress could induce cell apoptosis or cell death in many tumor categories, including breast cancer [4], neuroectodermal tumor [5], HCC [6], etc. ER stress could activate unfolded protein response (UPR) whose signaling network consists of three stress sensors, namely protein kinase RNA like ER kinase (PERK) or RNA-dependent protein kinase-like kinase, inositol-requiring enzyme 1α (IRE1α), and activating transcription factor 6 (ATF6) [7]. In cancer cells, UPR could be activated by exposure to hypoxia, oxidative stress and nutrient starvation. The tumor microenvironment usually features hypoxia and high content of reactive oxygen species (ROS) because of the highly active proliferation and metabolism status of cancer cells. Hypoxia and ROS could direct PERK activation, which in turn activates eukaryotic translation initiation factor 2α (eIF2α) which would induce the expression of activating transcription factor 4 (ATF4) for regulating redox homeostasis and metabolic homeostasis [8]. However CCAAT/enhancer-binding protein-homologous protein (CHOP, also known as GADD153), a downstream element to ATF4 in the PERK pathway, induces apoptosis or cell death under intensive or prolonged ER stress conditions [9, 10]. Interestingly, activating transcription factor 3 (ATF3), a bZIP DNA-binding protein, is associated with CHOP and thus integrated into the PERK/eIF2α pathway under ER stress backgrounds [11]. Expression of ATF3 could be induced by ER stress and involved in regulation of cell apoptosis [12]. In the tumors, ATF3 might induce cell apoptosis or improve cell survival depending on tumor types. Currently, several studies have shown that ATF3 plays tumor suppressing roles in different cancer types, including colon cancer [11] and esophageal squamous cell carcinomas (ESCC) [13]. It’s also reported that the overexpression of ATF3 suppresses growth of HeLa cells [14]. Other data showed that niclosamide, an antihelminthic drug for treatment of tapeworm infections approved by FDA, has exhibited anticancer function in different tumor types, including leukemia, colon cancer, glioma, etc [15]. We hypothesized that niclosamide also has effective function in anti-HCC. In this study, We demonstrated a new mechanism of niclosamide as anti-cancer with HCC cells.

## Methods

### Cell culture and drug treatment

HepG2 and QGY7701 cell lines were obtained from the Cell Bank of Shanghai Institute of Cell Biology (Chinese Academy of Sciences, Shanghai) and maintained in DMED (Hyclone, Logan, UT,) high glucose medium, supplemented with 10 % FBS (Gibco, NY). All cells were cultured in humidified 37 °C incubator supplied with 5 % CO2. Niclosamide (Sigma-Aldrich, St. Louis, MO) was dissolved in DMSO (Sigma-Aldrich, St. Louis, MO). To explore the regulation of niclosamide upon signal pathways, cells were seeded in 6-well plate and incubated for 24 h. Then cells were fed with fresh medium containing different concentrations of niclosamide or DMSO only as control. After 24 h of niclosamide treatment, cells was lysed with 1 % SDS lysis buffer (1%SDS,25 mM EDTA, 45 mM Tris-HCl, ph6.5) for western analysis, and total RNA was isolated directly with Trizol reagent (Life technologies, CA). In order to block ER stress, GSK2606414 (Selleck Chemicals, Houston, TX) was used to pretreat cells for 1 h before cells treated with niclosamide.

### DNA constructs and lenti-virus packaging

Oligo of ATF3 shRNA was synthesized and inserted in pGV298 lentiviral vector (GeneChem, Shanghai). The ATF3 target sequence was 5’-GCAAAGTGCCGAAACAAGA-3’ according to as described in publication [16]. The control GFP shRNA plasmid was purchased from GeneChem inc (GeneChem, Shanghai). The lentiviral vector is cotransfected with pVSV-G, pRev, pTat and pGag-pol (Gifts from Dr. Cheng, UMCM, Baltimore) in order to produce lentivirus particles in HEK293T cells with lipfectimin2000 (life technologies, CA) and supernatant were collected and were used to transduce HepG2 cells.

### Stable ATF3 knockout HepG2 cell line

HepG2 cells were planted to 6-well plate and incubated overnight. Cells were fed with medium contained ATF3 shRNA lentivirus particles and 10 μg.ml^−1^ polybrene(Santa Cruz, CA) which could improve the efficiency of lentivirus transduction. The medium was changed after 24 h of lentivirus transduction. Positive cells were selected with 10 μM puromycin (Santa Cruz, CA) and efficiency of ATF3 knockout was analyzed with western blotting.

### Cell viability assay

HepG2 and QGY7701 cells were seeded to 96-well plates and incubated overnight. Cells were fed with fresh complete medium containing different concentrations of niclosamide every 24 h and maintained for 3 days. Then cell viability was analyzed with CCK8 kit (Dojindo, Japan). Data were normalized with control group and presented as average ± SD. For the analysis of cell viability in ATF3 knock-down HepG2 and control cell lines, HepG2-ATF3^KD^ and HepG2-control cells were planted in 96-well plates. Cells were treated with niclosamide or DMSO and cell number was counted every day for three days. Data was presented as average ± SD. Student t-test was used in statist analysis.

### Terminal deoxynucleotidyl transferase-mediated dUTP nick end labeling (TUNEL) assay

TUNEL assay was performed to analyze cells apoptosis induced by niclosamide. HepG2 and QGY7701 cells grown on polylysine-coated cover slides were fixed with 4 % Paraformalclehyde after 24 h of 10 μM niclosamide treatment. TUNEL APO-GREEN detect kit (biotool, Shanghai, China) was used in DNA labeling according to manufacturer’s instruction. Images were taken by NIKON fluorescence microscope (Nikon, Japan).

### Flow cytometry

HepG2 and QGY7701 cells were treated with niclosamide, niclosamide/GSK2606414 or DMSO as control. Cells were harvested at 24 h of drug treatment and washed twice with cold PBS on ice. Cell pellets were resuspended with PBS and FITC-Annexin V Apoptosis Detection Kit (Wanlei Bio, Shenyang, China) was used to stain cells according to manufacturer’s manual book. Cells apoptosis were analyzed with flow cytometer (BD Bioscience, US).

### RNA preparation and quantitative real-time PCR

Total RNA was extracted with Trizol reagents (Life technologies, CA) according to manufacturer’s instruction. Reverse transcription and quantitative Real-time PCR (qRT-PCR) were performed as previously described [17]. For detecting gene expression induced by niclosamide, ATF3 and ER stress associated genes were selected for qRT-PCR and GAPDH was used as control. All primers were synthesized by Life technologies company (Shanghai, China) and the sequences were listed in Table 1. 2 μg total RNA was used as template to synthesize the first strand of cDNA with M-MLV reverse transcriptase (Takara, Dalian, China) in a 20 μl reaction system. PCR reactions were carried out with 200x diluted cDNAs, 100 nmol of each primer, and SYB Premix Ex Taq II (TaKaRa, Dalian, China) in a 20 μl reaction system. qRT-PCR reactions were performed with ABI (life technologies, CA) and PCR parameters involved the following steps: 95 °C for 5 min, 1 cycle; 94 °C for 5 s and 60 °C for 30 s, 40 cycles. Final data were normalized with GAPDH and presented as ratio to control.

**Table 1:** Primers for qRT-PCR

Gene Accession	Gene ID	Primer	Sequence
NM_001030287	ATF3	Forward(5’-3’)	CGAAGACTGGAGCAAAATG ATG
		Reverse(5’-3’)	CATCCAGGCCAGGTCTCTGCCTCAG
NM_001675	ATF4	Forward(5’-3’)	TGGACTTCGAGCAAGAGATG
		Reverse(5’-3’)	AGGAAGGAAGGCTGGAAGAG
NM_001195053	DDIT3(CHOP)	Forward(5’-3’)	TGCTTTCAGGTGTGGTGATG TATG
		Reverse(5’-3’)	AATCAGAGCTGGAACCTGAGGA
NM_004836	EIF2AK3(PERK)	Forward(5’-3’)	CTTATGCCAGACACACAGGA CAA
		Reverse(5’-3’)	TCCATCTGAGTGCTGAATGGAATAC
NM_007348	ATF6A	Forward(5’-3’)	GCCGCCGTCCCAGATATTA
		Reverse(5’-3’)	GCAAAGAGAGCAGAATCCCA
NM_001433	ERN1(IRE1A)	Forward(5’-3’)	ATTGTGTACCGGGGCATGTT
		Reverse(5’-3’)	TTCTCCGTGCAGAAGTAGCG
NM_001256799	GAPDH	Forward(5’-3’)	CTCAGACACCATGGGGAAG GTGA
		Reverse(5’-3’)	ATGATCTTGAGGCTGTTGTCATA

### Western immunoblotting

Western immunoblotting was performed as previously described [15]. The primary antibodies used in this study were anti-cleaved caspase 3 and anti- phospho-eIF2α (Ser51) (Cell signaling tech, Danvers), anti-GAPDH (Proteintech, Chicago), anti-ATF4 (Wanlei Bio, Shenyang, China), anti-GADD153/CHOP (Wanlei Bio, Shenyang,China), anti-ATF3, anti-eIF2α, anti-pPERK and anti-PERK (Santa Cruz biotech, Santa Cruz). HRP conjunct secondary Antibodies were purchased from Jackson immune Research-laboratories (Western Grove, PA). The PVDF membrane was purchased from Millipore (Millipore, Billerica) and ECL substrate was purchased from Thermo Fisher (Thermo Fisher, Waltham).

### Immunofluorescence staining and confocal microscopy

HepG2 and QGY7701 cells were grown on polylysine-coated cover slides and were fixed with cold methanol after 24 h of 2 μM niclosamide treatment. The cells were incubated in block buffer (3%BSA in TBST) at room temperature (RT) for 30 min. Then, anti-ATF3 or anti-GADD153/CHOP antibodies was diluted to 1:100 in blotting buffer (1 % BSA in TBST with 0.3 % TritonX-100) and were used to blot cells overnight at 4 °C. After the cells were washed three times with TBST, Alex®488-goat anti-rabbit IgG (Life technologies, CA) were used to incubate cells at RT for 1 h. Cell nucleus was stained with Hoechst 33342 (Sigma-Aldrich, St. Louis, MO). Confocal images were taken with Leica SP8 confocal microscope (Leica, Wetzlar, Germany).

### Statistical analysis

All calculations and statistical analyses were performed with Excel software (Microsoft, WA). Student t test was used in comparing two groups in experiments. All data were presented as average ± SD. All tests were two-sided, and *P* values less than 0.05 were considered to be statistically significant.

## Results

### Niclosamide suppressed cells growth by inducing ER-stress in HCC cells

**Fig. 1:**
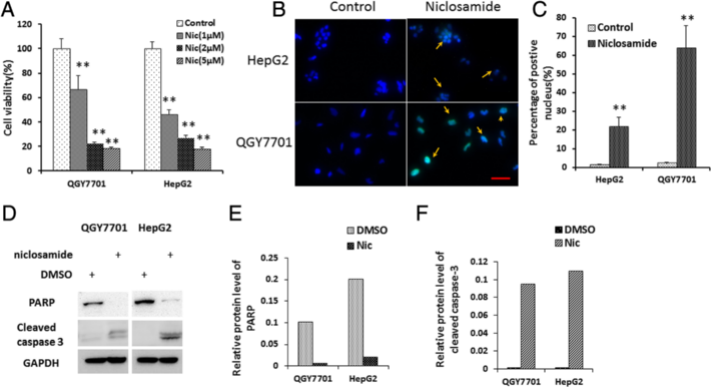
Niclosamide suppresses cell growth and induces cell apoptosis in hepatoma cells. **a** QGY7701 and HepG2 cells were treated with indicated concentrations of niclosamide and cell viability was analyzed using CCK-8 assay after 72 h of niclosamide treatment. Data from three independent experiments were normalized with DMSO control cells and presented as average ± SD. ** indicates *p* < 0.01. **b** QGY7701 and HepG2 cells were treated with 10 μM of niclosamide or equal volume of DMSO for 24 h. Cell apoptosis was analyzed with TUNEL assay, and apoptosis cell nuclei were labelled by FITC(Green) and all nuclei were stained with Hoechst 33342(Blue). Bar represents 50 μm. **c** Ratio of Nuclei of apoptosis cell was analyzed(*n* = 500). data Data was presented as average ± SD. **p < 0.01. **d** Cells were treated with 10 μM of niclosamide or equal volume of DMSO for 24 h. Cells were lysed with 1 % SDS lysis buffer and cleaved-caspase-3 and PARP protein level were analyzed with western blotting and GAPDH was used as loading control. **e** and **f** Results of western blotting was analyzed with Gel Image system software (Tanon) and data were presented as ratio of target protein to GAPDH in the form of grayscale value

**Fig. 2:**
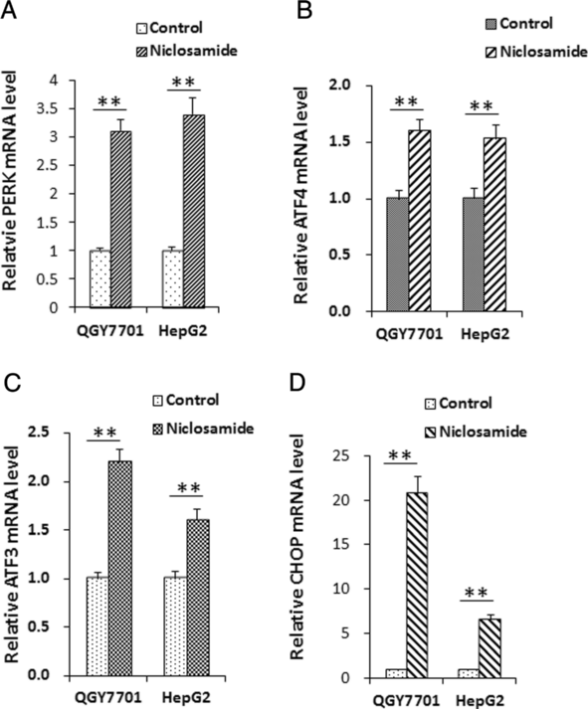
Expression of PERK signal pathway related genes was induced by niclosamide in hepatoma cells. QGY7701 and HepG2 cells were harvested and total RNA was extracted post treatment with 10 μM niclosamide in the medium for 24 h. **a **Expression level of PERK and its downstream genes, **b** ATF4, **c** ATF3 and **d** CHOP, were analyzed with qRT-PCR. Data were normalized with control group and presented as change-fold. All experiments were repeated for at least three times. ** indicates *p* < 0.01

Niclosamide significantly suppressed HCC growth in vitro as indicated by results of cell viability assay (Fig. 1a, b). The results of western blotting showed that niclosamide remarkably activated caspase-3 active and level of the poly ADP-ribose polymerase (PARP), a substrate of activated caspase-3, in niclosamide treatment cells was significantly less than in control cells (Fig. 1c, d, e). These data demonstrated activity of inducing apoptosis in hepatoma cells. To investigate the role of in ER-stress, the transcription levels of PERK, ATF6 and IRE1α, which are expressed specifically under the background of ER-stress, were analyzed using qRT-PCR. Interestingly, mRNA level of PERK but not ATF6 or IRE1α was significantly upregulated by niclosamide in both of HepG2 and QGY7701 cells (Fig. 2a).

**Fig. 3:**
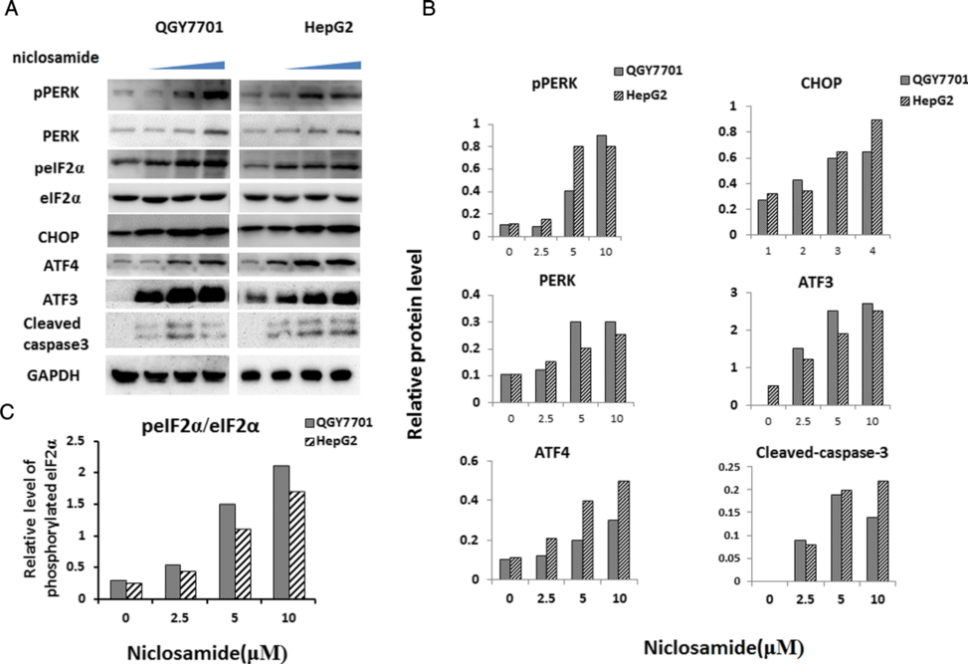
Niclosamide induced PERK activation and the expression of PERK downstream genes in hepatoma cells. **a** QGY7701 and HepG2 cells were planted in 6-well plates and cultured overnight. Cells were fed with fresh complete DMEM medium (10%FBS) with indicated concentration of niclosamide or DMSO. Cells were harvested and lysed with 1 % SDS lysis buffer after 24 h of niclosamide treatment. 30 μg of total protein was seperated by SDS-PAGE for immune-blotting. Commercial primary antibodies were used to probe target protein or phosphorylated protein on membrane. Signal of western blotting was captured by Tanon Gel Image system. **b** Results of western blotting were analyzed with Gel Image system software (Tanon) and data were presented as ratio of target protein to GAPDH in the form of grayscale value. **c** Relative phosphorylated eIF2α level was analyzed with western blotting and data were presented as ratio of phosph-eIF2α to total eIF2α in the form of grayscale value

ATF4 and CHOP are the most important downstream genes in the PERK-eIF2α pathway and modulate cell apoptosis [9]. Therefore, the expression of ATF3, ATF4 and CHOP were analyzed with RT-PCR and results showed that all of their mRNA levels were remarkably increased after niclosamide treatment (Fig. 2b, c, d). It’s also shown in our study that CHOP mRNA level was increased by over 20 times. To identify whether PERK pathway is activated by niclosamide, different doses of niclosamide was used to treat hepatoma cells and certain protein levels were analyzed with western blotting. We found protein levels of ATF4, ATF3 and CHOP, which are important transcription factors of the PERK pathway, were significantly increased in a dose dependent manner in accordance with the elevation of PERK protein level (Fig. 3a, b). In turn, phosphorylation of eIF2α was enhanced by active PERK (Fig. 3a, c). Interestingly, under normal conditions ATF3 level was low in HCC cells, but its elevation was more significant than ATF4 or CHOP (Fig. 3b). Our data suggested that niclosamide also activated caspase3 in both HepG2 and QGY7701 cells (Fig. 3a).

### Niclosamide increased nuclear accumulation of ATF3 and CHOP in HCC

**Fig. 4:**
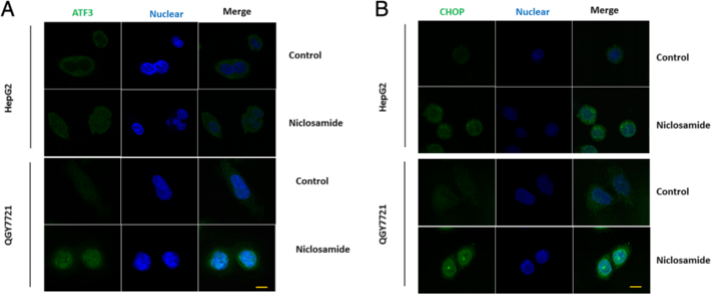
Niclosamide improved ATF3 and CHOP accumulation in the nucleus. QGY7701 or HepG2 cells were treated with 5 μM niclosamide or equal volume of DMSO in the medium for 24 h. Cells were immunostained using (**a**) anti-ATF3 antibdy or (**b**) anti-CHOP as described in Material and methods. The scan bar represented 10 μm

ATF3 and CHOP are critical transcription factors in the PERK pathway and they should bind to DNA to regulate gene transcription. To investigate whether ATF3 and CHOP upregulated by niclosamide localized in nucleus of hepatoma cells, Anti-ATF3 and Anti-CHOP primary antibodies was used in immunofluorescence assays. The results showed that niclosamide increase both ATF3 and CHOP levels and increase their accumulation in the nucleus of HepG2 and QGY7701 cells (Fig. 4a, b). These results demonstrated that niclosamide might upregulate ATF3 and CHOP expression, and such expression products would localize in nucleus to exert their roles.

### Niclosamide induced apoptosis in the HCC suppressed by PERK inhibition

**Fig. 5:**
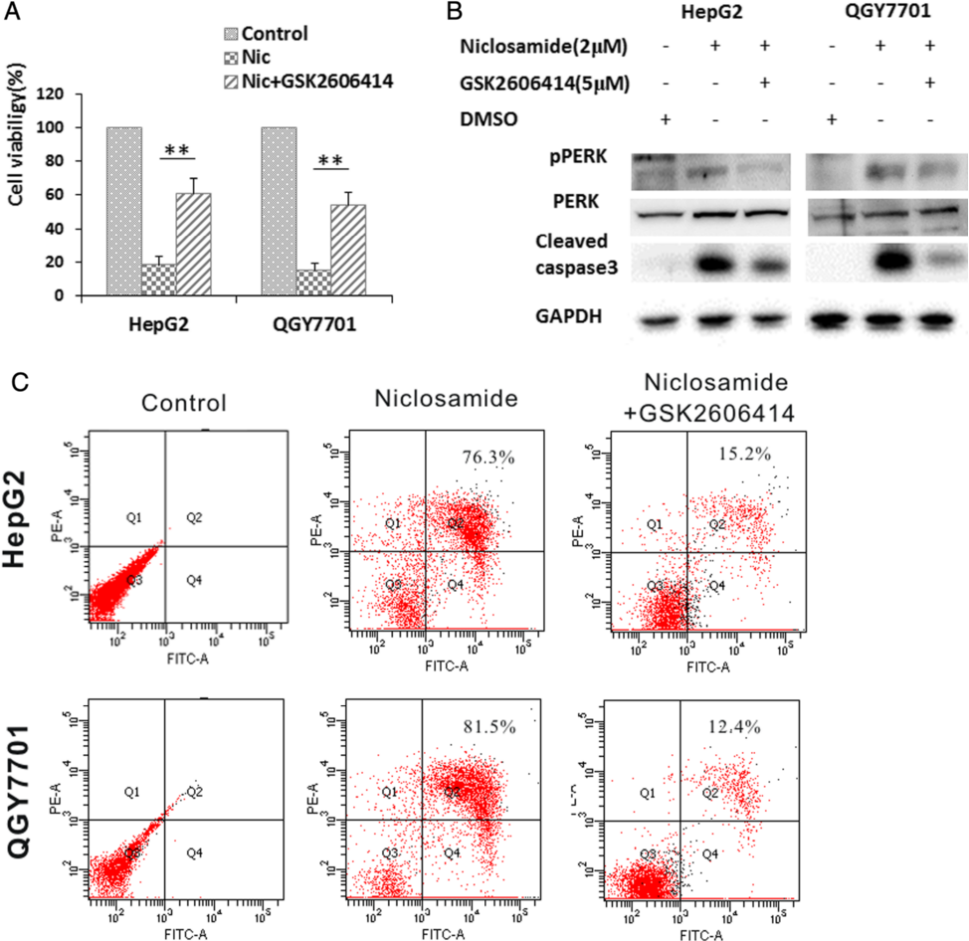
PERK inhibitor could atteneuate cell apoptosis induced by niclosamide in hepatoma cells. **a** QGY7701 and HepG2 cells were maintained with indicated drugs (2 μM of niclosamide or 2 μM of niclosamide + 5 μM of GSK2606414) in the medium for 72 h, DMSO was used as control. Cell viability was analyzed with CCK-8. **b** QGY7701 and HepG2 cells were harvested and lysed with 1 % SDS lysis buffer after 24 h of treatment with indicated drug. Western blotting was used to analyze levels of phospho-PERK, total PERK and cleaved caspase-3 in the cell lysate. **c** QGY7701 and HepG2 cells were treated with indicated drugs for 24 h. Then, cells were harvested and stained with FITC-Annexin V and PI for apoptosis assay with flow cytometry

Since PERK pathway was activated by niclosamide, we tried to investigate whether PERK pathway had its roles in the apoptosis induced by niclosamide. Our data showed that GSK2606414, an inhibitor of PERK pathway, could improve cell survival under niclosamide treatment (Fig. 5a). The results of western blot showed that GSK2606414 significantly suppress activation of caspase3 in HepG2 and QGY7701 cells (Fig. 5b). According to the cell flow cytometry assay data for cell apoptosis, GSK2606414 decrease percentage of apoptotic cells after niclosamide treatment (Fig. 5c). These evidences confirmed that PERK pathway contributes to cells apoptosis induced by niclosamide.

### ATF3 upregulation during cell apoptosis induced by niclosamide

**Fig. 6:**
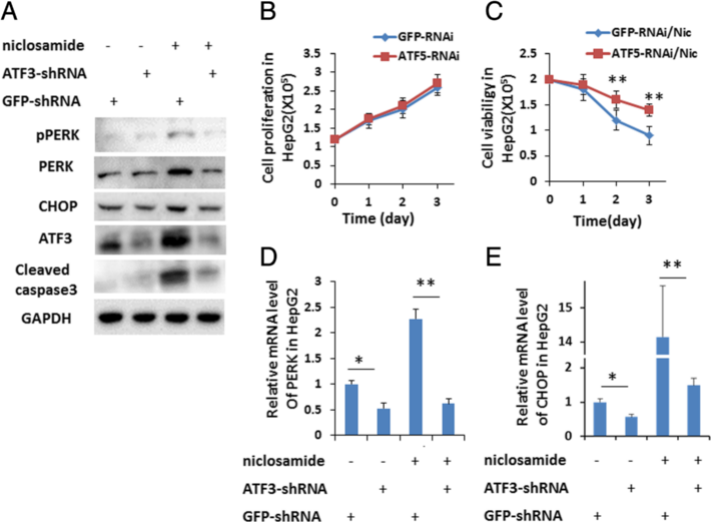
ATF3 was involved in modulation of cell apoptosis induced by niclosamide. **a** ATF3 knockdown HepG2 cell line (HepG2-ATF3^KD^) and control cell lines (GFP knockdown, HepG2-Control) was treated with indicated chemicals for 24 h and cells were harvested and lysed with 1 % SDS lysis buffer. 30 μg of total protein was seperated by SDS-PAGE for western blotting assay. Indicated primary antibodies were used for immunoblotting. HepG2-ATF3^KD^ and HepG2-control cells were planted in 96-well plates and maintained (**b**) without or with (**c**) 10 μM of niclosamide for 3 days. Cell number was countered every day. Data from three independent experiments were analyzed and presented as average ± SD. ** indicates *p* < 0.01. D and E, HepG2-ATF3^KD^ and HepG2-Control cells were treated with or without 10 μM of niclosamide for 24 h. Then, total RNA was extracted and cDNA was synthesized. Relative mRNA level of (**d**) PERK and (**e**) CHOP were analyzed with qRT-PCR. Data was presented as average ± SD. *p < 0.05,**p < 0.01

To verify the role of ATF3 in ER stress activated by niclosamide in HCC, we tried to down-regulate ATF3 in hepatoma cell lines with small hair RNA (ATF3-shRNA) targeting ATF3 gene and GFP gene (GFP-shRNA) as control. Our data showed there is no difference in cell proliferation between control cells and ATF3 knock-down (ATF3KD) cells in normal cell culture medium. However, ATF3KD cells had higher cell viability than control cells post niclosamide treatment (Fig. 6a). ATF3 knock-down might abrogate PERK and CHOP expression induced by niclosamide (Fig. 6b, c, d). In turn, ATF3 knock-down also reduced caspase3 activity induced by niclosamide (Fig. 6b). So our data suggested that ATF3 plays a central role in ER-stress activated and apoptosis induced by niclosamide in HepG2 cells.

## Discussion

In this study, our data suggested that niclosamide remarkably induced cell apoptosis (Fig. 1b) and increase expression level of PERK and its down-stream genes in HCCs (Fig. 2). It’s known that sustaining ER stress could sequentially activate IRE1α, ATF6 and PERK pathways [4, 8]. PERK/ATF4/CHOP pathway is one of most important pathways to induce cancer cell apoptosis in the UPR [18]. ATF3 is an important factor for stress response in cells and it interact with ATF4 and CHOP, these two transcription factors being PERK targeting genes under ER stress conditions [5, 9]. Current study demonstrated that ATF3 was involved in regulation of PERK function [19]. We further tried to reveal whether their protein levels are related with dose of niclosamide or not. Data of western blotting indicated that niclosamide upregulates PERK, ATF4, ATF3 and CHOP depending on the niclosamide concentration (Fig. 3). The results also demonstrated that caspase-3 is activated under niclosamide treatment (Fig. 3). Many studies had shown that niclosamide blocked cancer cells proliferation in a number of tumors types by suppressing the activities of several oncogenic pathways, including NF-kB, STAT3, notch and Wnt [20-22]. It also increases the level of reactive oxygen species (ROS) in acute myelogenous leukemia cells and enhances sensitivity to ROS in lung cancer cells [23]. Recently, it was identified that niclosamide could suppress HCC proliferation by blocking Wnt pathway [24]. Interestingly, we found that PERK pathway should play a critical role in HCC apoptosis induced by niclosamide. The apoptosis-inducing role of niclosamide in HCC could be abrogated by PERK inhibitor, GSK2606414 (Fig. 5). Several studies have suggested PERK/ATF4/CHOP pathway being involved in regulation of cells apoptosis and death in HCC [6, 25, 26].

**Fig. 7:**
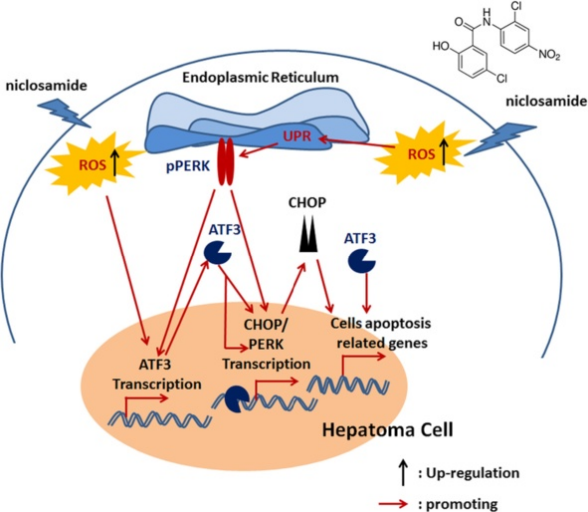
A model of niclosamide inducing cell apoptosis through ATF3-dependent PERK activation in hepatoma cells. In hepatoma cells, niclosamide could induce ROS which in turn activates the PERK pathway. Both activated PERK pathway and ROS could enhance ATF3 expression. PERK pathway and ATF3 directly regulate CHOP expression. ATF3 and CHOP would induce cells apoptosis. In addition, ATF3 also regulates PERK pathway through modulating PERK expression

Current studies have demonstrated that ATF3 plays an integral role in the PERK-eIF2α pathway in regulation of downstream gene expression when ER-stress is activated [27]. ATF3 is integrated in the PERK pathway, and its expression is regulated by PERK [19]. In the PERK pathway, ATF3 also modulates CHOP expression and regulates eIF2α activation by negative feedback [27]. In clinical HCC samples, the expression level of ATF3 is significantly low [28]. It’s also reported that ATF3 expression was low in HepG2 and QGY7701 cells. Niclosamide not only increases ATF3 mRNA and protein levels in HCCs, but also stimulates ATF3 accumulation in nucleus of HepG2 or QGY7701 cells (Fig. 4a). Because ATF3 functions in regulation of cancer cell survival and apoptosis depending on tumor types, its function in HCC was still unclear. Our data showed that downregulation of ATF3 expression level in HepG2 could remarkably abrogate effects of niclosamide on cell viability and activate caspase-3 level (Fig. 6a, b, c). Especially, PERK and CHOP expression levels were lower in ATF3 knock down cells than wild type cells either with or without niclosamide treatment (Fig. 6d, e). Several observations suggested that ATF3, an integrated player coordinating the expression of ER stress response related genes, directly regulates target gene expression which induce cell death or apoptosis in p53-dependent or independent ways in cancer cells under ER stress [16, 19]. It represses cancer-related chemokine expression and regulates cancer cells micro-environment under ER stress [11]. In our study, expression of ER stress target genes was repressed in an ATF3-absent condition in HepG2 and QGY7701 cells. Our study demonstrates a series of crucial roles of ATF3 in PERK/CHOP pathway activation and cells apoptosis induction resulting from niclosamide treatment in HCC (Fig. 7).

## Conclusion

Our study reveals that niclosamide activates PERK and up-regulates both of ATF3 and CHOP expression in HCCs. The function of niclosamide could be abrogated by PERK inhibitor and suppression of ATF3 expression. Especially, ATF3 upregulates PERK and CHOP level in HCCs being exposed to niclosamide. Our data indicates that ATF3 plays a central role in the induction of cell apoptosis by niclosamide in HCC.
